# Nitrogen signals and their ecological significance for seed germination of ten psammophilous plant species from European dry acidic grasslands

**DOI:** 10.1371/journal.pone.0244737

**Published:** 2021-01-04

**Authors:** Mateusz Wala, Jeremi Kołodziejek, Jacek Patykowski

**Affiliations:** 1 Department of Geobotany and Plant Ecology, Faculty of Biology and Environmental Protection, University of Lodz, Łódź, Poland; 2 Komandorska, Łódź, Poland; Chinese Academy of Forestry, CHINA

## Abstract

The presented study evaluated effects of potassium nitrate (KNO_3_), ammonium nitrate (NH_4_ NO_3_) and ammonium chloride (NH_4_Cl) on the germination-related characteristics of 10 species from European dry acidic grasslands. Germination was studied under controlled laboratory conditions. The seeds were subjected to KNO_3_, NH_4_ NO_3_ and NH_4_Cl in four doses (1, 10, 50 and 100 mM) and to distilled water. Final germination percentage, index of germination velocity and index of germination synchrony were determined. Content of nitrogen in the soil probed from the site of seeds collection was also analyzed. Significant effects of type of the nitrogen compounds and their concentrations were observed. High concentrations of nitrogen-containing salts inhibited completion of germination in almost all species. *Helichrysum arenarium* and *Hypericum perforatum* showed preference for NH_4_^+^ over NO_3_^‒^, whereas *Arnoseris minima*, *Alyssum montanum*, *Jasione montana* and *Spergula morisonii* showed preference for NO_3_^‒^ over NH_4_^+^. *Centaurea scabiosa*, *C*. *stoebe* and *Hypochaeris radicata* had no preference and wide tolerance to the type of nitrogen-containing compound. *Echium vulgare* showed differential response hard for interpretation. *A*. *montanum* and *J*. *montana* showed stenotopic behavior in terms of nitrogen-related conditions. It is proposed that nitrogen-rich soil gaps favor establishment of more nitro-tolerant plant species (e.g. *C*. *scabiosa*, *C*. *stoebe* and *H*. *radicata*) as compared to nitrogen-poor ones.

## Introduction

Dry acid grasslands typically occur on free-draining soils overlying acid rocks or superficial deposits such as sands and gravels, which were deposited mostly during and after the last ice age [1]. High surface temperatures, low water storage, low nutrient contents, low organic matter content and litter cover on sandy substrate are the main characteristics of this habitat [2, 3]. The psammophilous grassland from *Spergulo morisonii-Corynephoretum canescentis* (Tx. 1928) Libb. 1933 (syn. *Corniculario aculeatae-Corynephoretum canescentis* Steffen 1931) [4] is a most common plant association formed on those poor and acidic sands. They are composed of pioneer plant species with wide and low/specialized environmental requirements. According to previous studies [4], the *Spergulo-Corynephoretum* is the only association within the *Corynephorion canescentis* Klika 1931 (order *Corynephoretalia canescentis*, Klika 1934, class *Koelerio-Corynephoretea* Klika in Klika & Novak 1941). The most characteristic herb and grass species for this association are *Achillea millefolium* L., *Corynephorus canescens* (L.) P. Beauv., *Helichrysum arenarium* (L.) Moench, *Pilosella officinarum* Vaill. (syn. *Hieracium pilosella* L.), *Hypochaeris radicata* L., *Jasione montana* L., *Rumex acetosella* L., *Scleranthus perennis* L., *Spergula morisonii* Boreau, *Teesdalia nudicaulis* (L.) W. T. Aiton and *Veronica dillenii* Crantz [4, 5].

Re-colonizing dynamics in dry acidic grasslands are strongly determined by regeneration characteristics of each species, such as life cycle, dispersal abilities and germination pattern [2]. Thus, it can be expected that at least some vegetation pattern changes observed in field can be explained by differences in germination requirements. Low water holding capacity of the soil and possibly soil nitrogen (N) status are the main factors limiting completion of germination and seedling survival in this kind of environments [6]. Total N concentrations recorded in the soils of *Spergulo-Corynephoretum* vary between 0.01 and 0.08% of soil dry weight (SDW) (equal to 100–800 mg kg^-1^ SDW [2]) which suggests its poor nutritional quality. Regarding N type, there are two major mineral forms available to plants, i.e. ammonium (NH_4_^+^) and nitrate (NO_3_^‒^). On inland sand dunes, NH_4_^+^ soil concentration is typically higher than that of NO_3_^‒^ [7–9]. Low net N mineralization and greater concentration of NH_4_^+^ than NO_3_^‒^ are connected with N circulation process and result from halted nitrification process in pioneer sand-settled communities [9]. Thus, acidic soils provide NH_4_^+^ as the predominant chemical form of N and, interestingly, this form of N is preferred by acidophilous plant species, while more or less calciphilous species prefer NO_3_^‒^ [10]. However, this is totally opposite to molecular basis of N uptake, where easier scavenging of NO_3_^‒^ by plants can be observed in acidic soils due to greater availability of H^+^ for NO_3_^‒^/H^+^ symport mechanism [11].

The composition and diversity of species as well as primary productivity of terrestrial ecosystems are strongly affected by the sources of limiting nutrients, e.g. N [12]. It is an essential macronutrient for plants and is required for many primary and secondary metabolic processes. Regarding germination process, N may modulate germination in many plant species, both alone and in combination with other abiotic factors, e.g. changing temperature or light-dark cycle [13–15]. While the stimulating role of NO_3_^‒^ on completion of seed germination was well recognized [15, 16], mode of action of NH_4_^+^ is more enigmatic. NH_4_^+^-driven alternations of seed metabolism during germination are associated with decelerated mobilization of seed resources [17] which may reduce germination-related parameters [18] however NH_4_^+^-tolerant plants are less susceptible than intolerant ones. These harmful effects are related to toxic action of NH_4_^+^ [19], especially in tissues with not overdriven metabolism, where NH_4_^+^ cannot be directly incorporated into bio-molecules. It must be mentioned that besides N availability, pH is another major factor limiting plant growth on acid soils. As an example, pH of the soils on which dry inland grasslands (*Spergulo-Corynephoretum*) are settled ranges 3.6–6.8 [2]. As plants are able to absorb N in cationic (e.g. NH_4_^+^) and anionic (e.g. NO_3_^‒^) forms, uptake of nitrogen-containing compounds influences the pH in rooting substratum [20, 21]. It is known that pH values of 1–100 mM solutions of KNO_3_, NH_4_NO_3_ and NH_4_Cl are slightly different [14]. Therefore, uptake and utilization of N by plant seeds and then seedlings can lead to gradual depletion of N sources on a local scale which may shift the pH status and create microhabitats, especially in sandy soils lacking developed sorption complex and when species-characteristic N uptake pattern can be observed.

Only a few studies focused on the germination requirements of plant species in dry sandy grasslands [22, 23]. Differential effect of NO_3_^‒^ and NH_4_^+^ on plant growth and yield [18] as well as the importance of N for development and persistence of acidic dry grasslands were analyzed [2] however the comparative studies on influence of N type and dose on completion of germination are not very common in literature. It can be hypothesized that exposure of seeds to exogenous N-containing salts can significantly affect ability of seeds to complete germination. Furthermore, it can be expected that NH_4_^+^ and NO_3_^‒^ exert different effects on completion of seed germination. It is also not known if the studied plant species are similar in germination requirements. As an output of this study we intended to answer the following questions: 1) is nitrogen a factor controlling seed-based revegetation and 2) is the ability to complete germination under wide range of conditions species dependent. The hypotheses tested are that 1) nitrogen type and dose affect germination-related characteristics of the studied species and 2) responses to nitrogen type and dose vary among plant species.

## Material and methods

### Selection of species and collection of seeds

Seeds of 10 species (nomenclature follows The Plant List [24]) native to central Europe were used in this study: *Alyssum montanum* L., *Arnoseris minima* (L.) Schweigg. & Körte, *Centaurea stoebe* L., *C*. *scabiosa* L., *Echium vulgare* L., *Helichrysum arenarium* (L.) Moench, L., *Hypochaeris radicata* L., *Hypericum perforatum* L., *Jasione montana* L. and *Spergula morisonii* Boreau (syn. *S*. *vernalis* Willd.) (Table 1). These species are from 6 families (Brassicaceae, Asteraceae, Boraginaceae, Hypericaceae, Campanulaceae and Caryophyllaceae) with the majority from Asteraceae. The species we studied were selected based on their Ellenberg Indicator Values [25] for soil reaction (R, Table 1). All species we studied are light-demanding, require relatively high temperature, medium soil moisture and have low N indicators of ecological behavior, typical of N low habitats (Table 1) [25]. The species we selected were among the most representative and abundant in the patches of *Spergulo-Corynephoretum* located in the eastern village periphery of Niewiesz in central Poland (19°83’N, 10°83’E at 149 m a.s.l.). The studied plant community is settled on Entic Podzol soil. The local climate is temperate (annual precipitation of 587.2 mm and a temperature range of –2.5 to 22.4°C [26]), and the seasons are clearly differentiated. Meteorological data based on 10-year observations (2000–2010) indicated that the mean annual temperature was 8.8°C.

**Table 1:** List of the studied species, their seed characteristics and habitat preferences of mature plants.

Species	Abbreviation	Family	Growth form	Seed size [mm]^a^	Seed weight [mg]^b^	Ellenberg Index^c^					
						L	T	K	F	R	N
*Alyssum montanum* L.	Amo	Brassicaceae	Perennial herb	1.5–1.9 x 1.1–1.3	0.368 ± 0.028	9	6	4	2	7	1
*Arnoseris minima* (L.) Schweigg. & Körte	Ami	Asteraceae	Annual herb	1.6–2.2 x 0.6–0.7	0.215 ± 0.019	7	6	2	4	3	3
*Centaurea scabiosa* L.	Csc	Asteraceae	Perennial herb	4.5–5.0 x 2.0–2.2	3.095 ± 0.133	7	0	3	3	8	4
*C*. *stoebe* Tausch	Cst	Asteraceae	Biennial herb	2.5–3.0 x 1.2–1.4	1.023 ± 0.034	8	7	6	2	8	3
*Echium vulgare* L.	Evu	Boraginaceae	Biennial herb	2.4–2.8 x 1.5–1.8	1.940 ± 0.104	9	6	3	4	8	4
*Helichrysum arenarium* (L.) Moench	Har	Asteraceae	Perennial herb	0.9–1.1 x 0.4–0.5	0.043 ± 0.005	8	6	7	2	5	1
*Hypericum perforatum* L.	Hpe	Hypericaceae	Perennial herb	1.0–1.2 x 0.5–0.6	0.060 ± 0.008	7	6	5	4	6	3
*Hypochaeris radicata* L.	Hra	Asteraceae	Perennial herb	5.0–10.0 x 0.5–0.6	0.578 ± 0.038	8	5	3	5	4	3
*Jasione montana* L.	Jmo	Campanulaceae	Annual/biennial herb	0.6–0.8 x 0.2–0.3	0.018 ± 0.005	7	6	3	3	3	2
*Spergula morisonii* Boreau	Smo	Caryophyllaceae	Annual herb	1.3–1.7 x 1.3–1.6	0.293 ± 0.024	9	5	4	3	0	2

Nomenclature of studied taxa follows the Plant List [24]

^a^seed size follows literature [28]

^b^seed weight measured in this study (determined by weighing 100 or 500 air-dried seeds, expressed as mean (SD); n = 4)

^c^habitat preference of mature plants follows 1–9 point scale proposed by Ellenberg [25]. L–light requirements ranging 7–9, where 7 denotes semi-light conditions (c.a. 30% of relative illumination) and 9 denotes full light conditions (> 50% of relative illumination); T–temperature requirements ranging 5–7, where 5 denotes species preferring moderately cool to warm conditions (characteristic of montane and submontane conditions, mostly southern Fennoscandia) and 7 denotes species preferring warm conditions (characteristic of North European Plain); K–continentiality requirements ranging 2–7, where 2 denotes atlantic conditions and 7 denotes subcontinental conditions; F–soil moisture requirements ranging 2–5, where 2 denotes dry and extremely dry soils and 5 denotes moist soils; R–soil pH requirements ranging 3–8, where 3 denotes slightly acidic to slightly basic soils and 8 denotes average basic soils originating from limestones. N–nitrogen availability requirements ranging 1–4, where 1 denotes extremely infertile soils and 4 denotes slightly and intermediately fertile soils; 0 –indifferent behavior, wide amplitude or unequal behavior in different areas.

Mature seeds of all species were hand-harvested at the end of the summer of 2016, coinciding with their dispersal period. The seeds were taken from at least 10 individuals of one single, large and representative population. The seeds were collected on privately-owned property, where no specific permissions are required. The seeds were stored dry in paper bags at 20–22°C (relative humidity of 30%) for two weeks and then used for experiments within one month. Stratification of the seeds was not conducted due to the fact that fresh seeds of the species we studied are able to efficiently complete germination [27]. At this stage, the seeds were inspected under binocular magnifying glass; all malformed, wrinkled, damaged or discolored ones were removed from the seed pool. After inspection, the seeds were weighed. Dimensions of the seeds were taken from the literature [28].

### General germination procedures

Ability of the seeds to complete germination was tested in glass Petri dishes (ø = 5 cm). The seeds of each species were mixed before use to fulfil the randomization requirement. The seeds (25 per one Petri dish) were placed on double-layer filter paper and moistened with 3 ml of the tested solutions. To maintain stable and homogenous conditions all dishes were sealed with parafilm. The moisture level was monitored daily and the seeds were re-watered when any loss was observed. Germination test was conducted under 16/8 h photoperiod (light was supplied with white fluorescent tubes with a photon flux density of 52 μmol m^−2^·s^−1^) at 23°C. Close to natural photoperiod was used, due to the fact that the seeds of the species we studied complete germination only under sufficient light conditions [27, 29–31]. The temperature of 23°C was listed as optimal for completion of germination for many herbaceous plant species from the temperate region (including wild-living rangeland species [29, 32] and species from dry acidic grasslands) and represents the mean temperatures at the Lodz Weather Station during June and July, when most seeds germinate in natural habitats. Furthermore, it allows direct comparisons of many species subjected to various chemical treatments [33, 34]. Experiments were repeated four times (n = 4). Due to the fact that completion of germination of the studied species reached plateau after 12 days in the preliminary test, it was recorded daily for 14 days. The seeds that completed germination were removed from the Petri dishes. An emerged radicle was a sign of completed germination. Germination-related traits were determined on the basis of the number of viable seeds. Dead seeds (with soft and brown embryo) were not included in the calculations.

### Treatments and experimental setup

The seeds were treated with 1, 10, 50 or 100 mM solutions of KNO_3_, NH_4_NO_3_ or NH_4_Cl. For control, distilled water was used (conductivity < 0.06 μS cm^-1^). Thus, the experimental design consisted of 13 experimental groups for each species. By such a range of nitrogen types and doses we intended to simulate conditions which can be observed in nature as well as to create extreme situations which cannot be encountered there. The seeds subjected to distilled water (0 mM of N) germinated under absolute non-N conditions which are very rare in the field. Treatment with 100 mM of N was used to simulate the conditions with exceeding concentration of N which is not likely in sand-settled plant communities. Solutions of 1, 10 and 50 mM simulated very poor (regarding N concentration) conditions, perturbed soil with exposed deeper layers (where N content is c.a. 0.02% kg^-1^ soil) and typical soil surface of sand soil, e.g. Podzol (where N content is c.a. 0.09% kg^-1^ soil), respectively. Furthermore, previous studies indicated that 0–100 mM range of solutions of N-containing salts is suitable for studies on germination requirements [35]. According to literature [14], pH values of the tested solutions are slightly different (Table 2).

**Table 2:** Acidity (pH) of water solutions of nitrogen-containing salts used in this study.

Nitrogen type	pH			
	1 mM	10 mM	50 mM	100 mM
KNO_3_	5.13	5.76	5.86	6.01
NH_4_NO_3_	5.50	6.36	6.80	6.80
NH_4_Cl	5.10	5.65	5.83	5.80

The data follows literature [14].

### Determination of germination related parameters

To estimate reactions of the studied species to nitrogen type and dose, final germination percentage (FGP), index of germination velocity (IGV, known as modified Timson’s index [36]) and index of germination synchrony (IGS, known as Z value [37]) were calculated. Higher values of FGP (where minimum is 0% and maximum is 100%) indicate greater ability to complete germination. Higher values of IGV (where minimum is 0 and maximum is 100) denote more rapid completion of germination. Higher values of IGS (where minimum is 0 and maximum is 1) indicate greater synchronization of germination. The germination-related parameters were calculated using the germinationmetrics package v. 0.1.3 [38] run in R software (v. 3.5.2, 64 bit version [39]) with the functions implemented therein.

### Analysis of the soil from the habitat of the studied species

The soil analyzed in this study was probed from the same site from which the seeds were collected. Four representative plots (1 × 1 m) were designated. Subsequently, one soil subsample (0–15 cm soil depth core; ø = 2 cm) from each plot was collected. All subsamples were pooled into a single bulk sample.

The soil analysis was conducted by Regional Chemical and Agricultural Station in Łódź according to the certified norms and procedures used in Poland. Soil pH_KCl_ was measured using the potentiometric method according to the PN-ISO 10390:1997 norm. Total N was determined by the titrimetric Kjeldahl method (procedure PB 49 ed. 2) and N-NO_3_^‒^ and N-NH_4_^+^ were determined using the continuous flow analysis (CFA) coupled with spectrophotometric detection (procedure PB 50 ed. 1).

### Statistical analysis

One-way ANOVA analyses were conducted do detect differences between groups. For further analysis, the conservative Bonferroni post-hoc test was used to detect differences between mean values. This test was selected because it allows to compare large numbers of groups without erroneous results of hypothesis testing. Differences were considered significant at *P* < 0.05. To estimate influence of the studied factors, two-way ANOVA and three-way ANOVA were used for analysis among each species and for analysis of all species together, respectively. All analyses were conducted using StatisticaTM v. 13.3 (Tibco Software Inc.).

## Results

### Germination-related parameters

**Fig 1:**
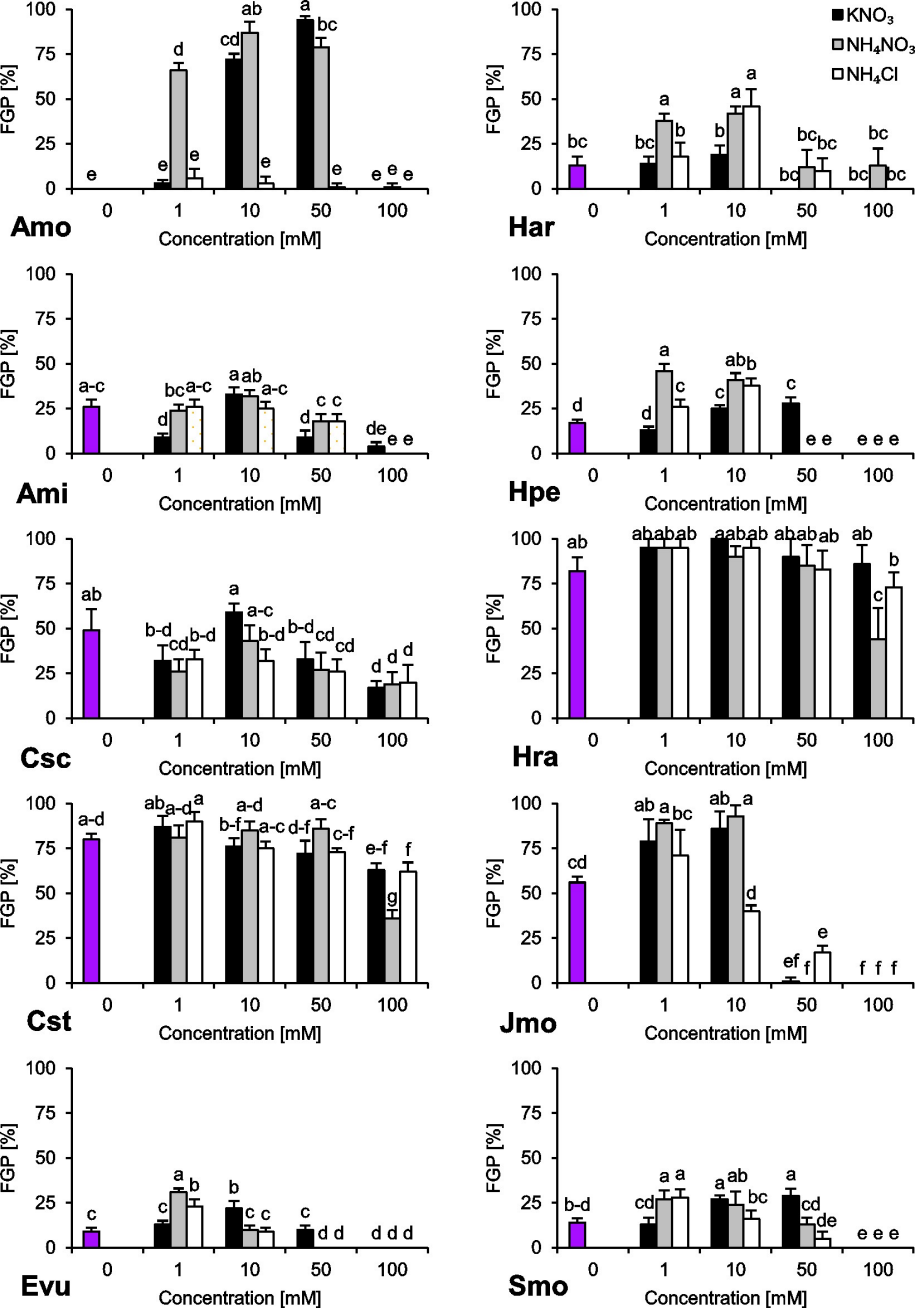
Final Germination Percentage (FGP) of ten studied species subjected to different nitrogen types and doses. The seeds were incubated at 23°C under a 16/8 h photoperiod for 14 days. Values presented are the mean (SD) (n = 4). Different letters indicate significant differences between groups (ANOVA, Bonferroni’s post-hoc test at *P* < 0.05).

**Fig 2:**
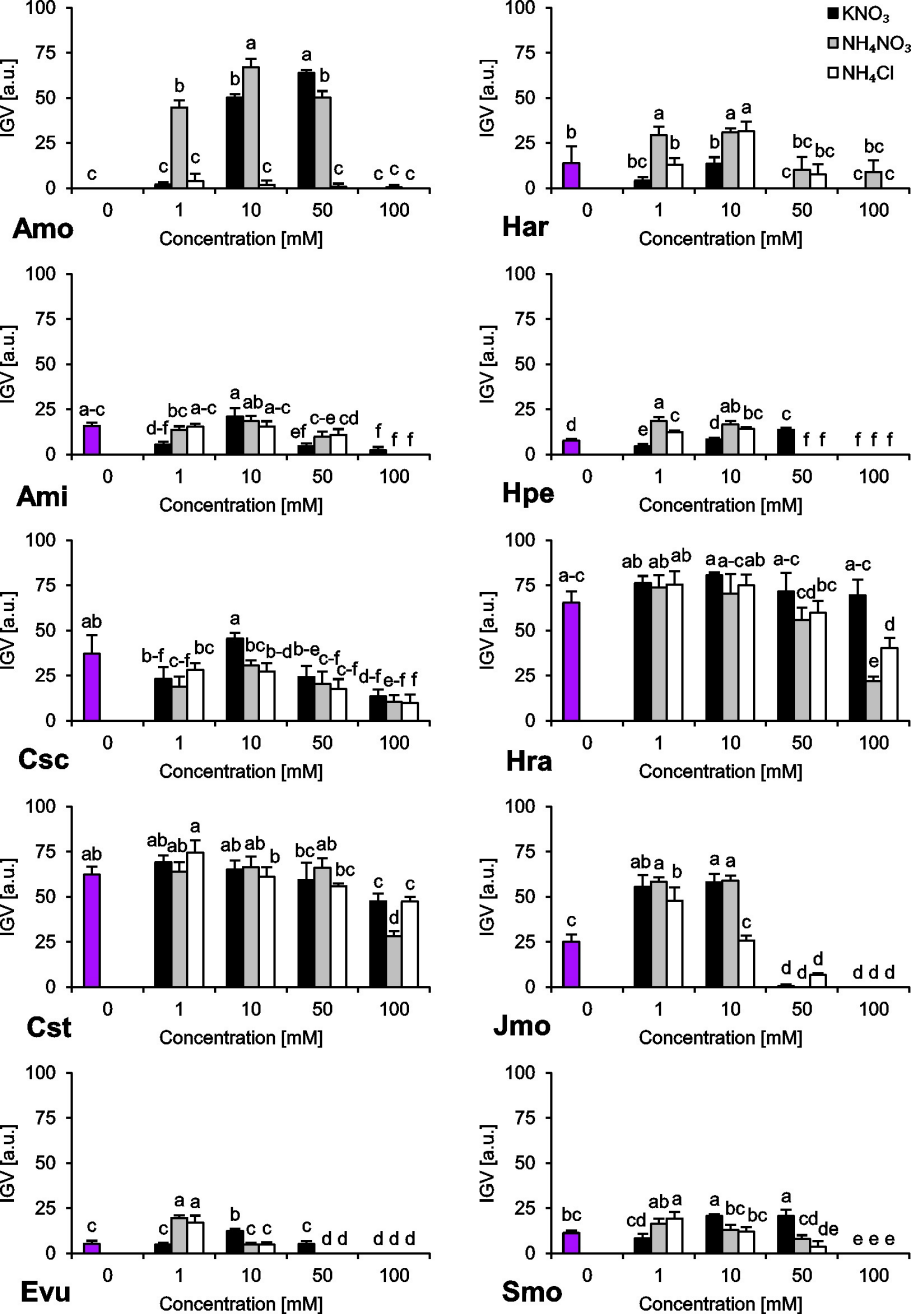
Index of Germination Velocity (IGV) of ten studied species subjected to different nitrogen types and doses. The seeds were incubated at 23°C under a 14 h photoperiod for 16/8 days. Values presented are the mean (SD) (n = 4). Different letters indicate significant differences between groups (ANOVA, Bonferroni’s post-hoc test at *P* < 0.05).

**Fig 3:**
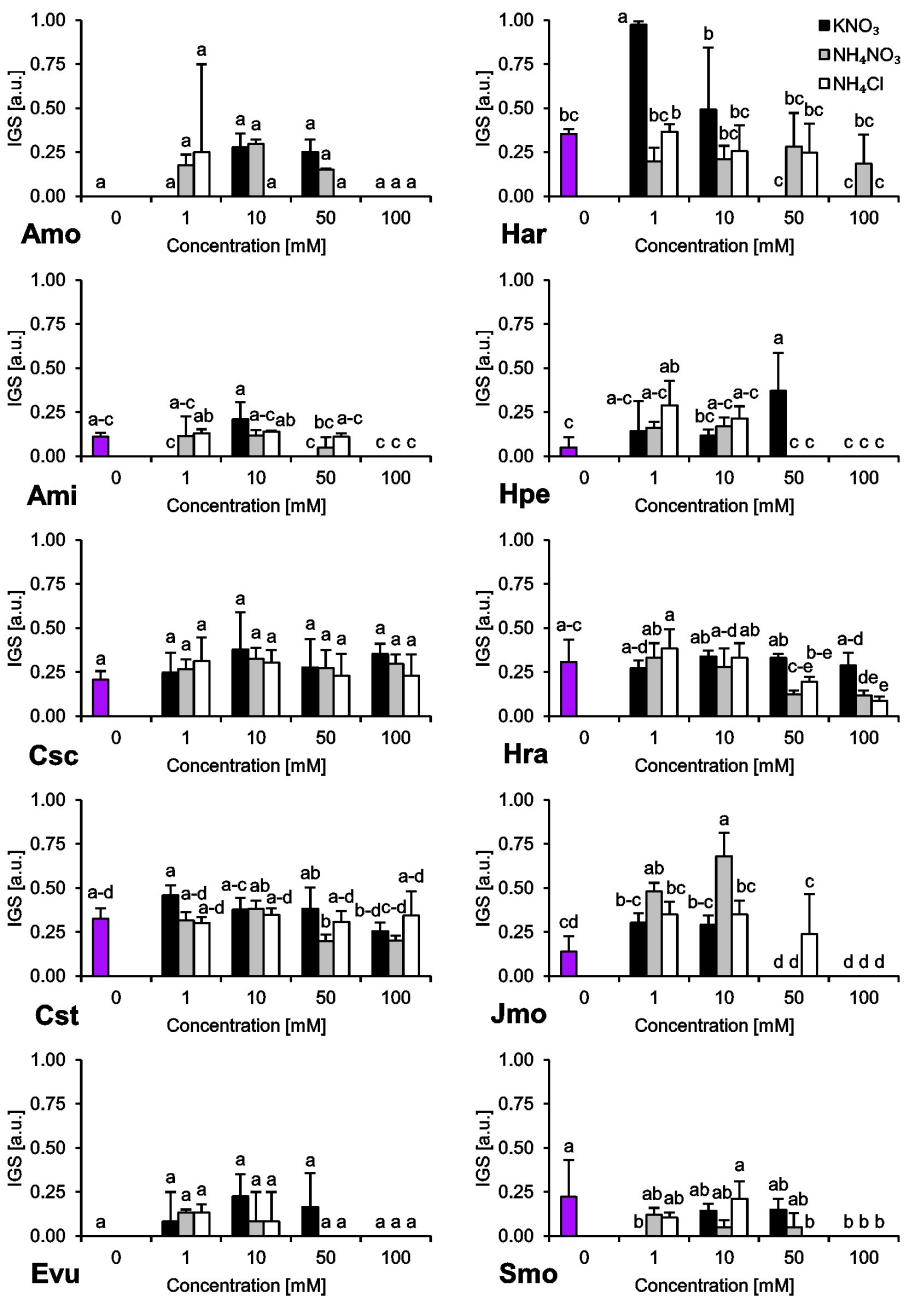
Index of Germination Synchrony (IGS) of ten studied species subjected to different nitrogen types and doses. The seeds were incubated at 23°C under a 16/8 h photoperiod for 14 days. Values presented are the mean (SD) (n = 4). Different letters indicate significant differences between groups (ANOVA, Bonferroni’s post-hoc test at *P* < 0.05).

The species we studied showed generally differential reactions to the examined nitrogen compounds and their concentrations. In the absence of any form of N, we observed a wide range of responses regarding final germination percentage (FGP). The seeds subjected to distilled water showed low FGP, ranging 9–26% (*A*. *minima*, *E*. *vulgare*, *H*. *arenarium*, *H*. *perforatum* and *S*. *morisonii*), medium FGP, ranging 49–56% (*C*. *scabiosa* and *J*. *montana*) or very high FGP, ranging 80–82% (*C*. *stoebe* and *H*. *radicata*) (Fig 1). Interestingly, we were unable to record any completion of germination of *A*. *montanum* seeds subjected to this treatment (Fig 1). Similar trend was observed for index of germination velocity (IGV) describing germination speed (Fig 2) and index of germination synchrony (IGS) indicating synchronization of germination (Fig 3).

Regarding preferences of N type as a germination stimulator, we were able to distinguish three major groups of plant species. The first group (*H*. *arenarium* and *H*. *perforatum*) preferred NH_4_^+^ over NO_3_^‒^ as a signal for completion of germination (Fig 1). The second group (*A*. *montanum*, *J*. *montana* and *S*. *morisonii*) is composed of species preferring NO_3_^‒^ over NH_4_^+^ as a stimulant for completion of germination (Fig 1). The third group (*C*. *scabiosa*, *C*. *stoebe*, *H*. *radicata*) is composed of species with no preference and wide tolerance to N type (Fig 1). *E*. *vulgare* and *A*. *minima* showed differential response, hard for interpretation.

Two of the studied species showed stenotopic behavior (Fig 1). In the case of *A*. *montanum*, a relatively narrow window for stimulation of germination process ranging 1–50 mM of NH_4_NO_3_ and 10–50 mM KNO_3_ was observed while *J*. *montana* was able to complete germination in solutions ranging 0–10 mM N.

Two-way ANOVA showed that N type and dose had significant influence on FGP and IGV (with very few exceptions of *C*. *stoebe* and *E*. *vulgare*) (Table 3). All interactions of N type and dose had significant influence on FGP and IGV of all species (Table 3). According to two-way ANOVA, N type significantly influenced IGS only in *C*. *stoebe*, *H*. *arenarium*, *H*. *radicata* and *J*. *montana*, whereas influence of N dose on IGS was insignificant only in *C*. *scabiosa* (Table 3). Interactions of N type and dose had no influence on IGS of *C*. *scabiosa* and *E*. *vulgare* (Table 3).

**Table 3:** Results of two-way ANOVA (*F* value) showing influence of nitrogen type and dose on Final Germination Percentage (FGP), Index of Germination Velocity (IGV) and Index of Germination Synchrony (IGS) of studied species.

Species	Nitrogen	Nitrogen	NT x ND
	Type (NT)^a^	Dose (ND)^b^	
	(*df* = 2)	(*df* = 3)	(*df* = 6)
	FGP		
Amo	1078.7***	716.7***	269.2***
Ami	9.1***	159.2***	14.2***
Csc	4.7*	24.3***	3.5**
Cst	1.6n.s.	89.0***	17.9***
Evu	10.0***	278.9***	46.8***
Har	34.3***	67.4***	5.2***
Hpe	24.0***	462.5***	112.0***
Hra	8.8***	21.5***	4.3**
Jmo	17.8***	500.1***	24.9***
Smo	7.1**	88.1***	19.6***
	IGV		
Amo	911.8***	610.7***	233.1***
Ami	3.9***	113.0***	9.9***
Csc	8.8***	46.2***	4.4**
Cst	3.0n.s.	68.6***	8.4***
Evu	0.9n.s.	239.6***	59.9***
Har	55.5***	70.4***	6.5***
Hpe	24.7***	468.0***	164.3***
Hra	30.4***	54.4***	8.4***
Jmo	36.2***	850.2***	33.7***
Smo	11.5***	107.8***	28.2***
	IGS		
Amo	1.7n.s.	3.7*	3.1*
Ami	3.2n.s.	22.5***	5.2***
Csc	0.6n.s.	1.0n.s.	0.5n.s.
Cst	7.8**	6.2**	3.6**
Evu	1.9n.s.	3.7*	1.3n.s.
Har	5.6**	21.6***	12.4***
Hpe	2.6n.s.	10.5***	7.5***
Hra	8.9***	18.8***	5.9***
Jmo	10.9***	75.8***	9.1***
Smo	1.1n.s.	18.1***	10.6***

^a^KNO_3_, NH_4_NO_3_ or NH_4_Cl

^b^1, 10, 50 or 100 mM solutions.

*P < 0.05

**P < 0.01

***P < 0.001, ns–not significant.

According to three-way ANOVA, all analyzed factors (species, N type and N dose) significantly affected the measured germination-related traits (Table 4).

**Table 4:** Results of three-way ANOVA (*F* value) showing effects of studied factors on germination-related parameters.

Factor	*df*	*F* values and significance		
		FGP	IGV	IGS
(S) Species^a^	9	1060.4***	1420.0***	49.7***
(NT) Nitrogen Type^b^	2	88.9***	80.2***	6.0**.
(ND) Nitrogen Dose^c^	3	879.5***	932.6***	89.5***
S x NT	18	51.0***	63.1***	3.6***
S x ND	27	69.6***	69.3***	6.6***
NT x ND	6	59.8***	63.5***	2.2*
S x NT x ND	54	18.9***	18.1***	6.1***

^a^ten studied species (see Table 1 for details)

^b^KNO_3_, NH_4_NO_3_ or NH_4_Cl

c1, 10, 50 or 100 mM solutions.

*P < 0.05

**P < 0.01

***P < 0.001, n.s.–not significant.

### Nitrogen status of the soil from the collection site of seeds

Soil analysis (Table 5) showed that acidity of the tested Entic Podzol was typical of this type of soil (pH_KCl_ = 5.7) and met the requirements of the studied plant community. Analysis also showed that the soil contained relatively low total N. Mineral forms of N (N-NO_3_^‒^ and N-NH_4_^+^) did not contribute substantially to the total N pool (0.99% of total N). Molar N-NH_4_^+^: N-NO_3_^‒^ ratio of 1.57 showed that N-NH_4_^+^ was a predominant N form in the studied soil.

**Table 5:** Nitrogen (N) status of Entic Podzol probed from site of seeds collection.

N-related	Entic Podzol (pH_KCl_ = 5.7)	
	Unit of measurement	
soil characteristic		
	[mg kg^-1^ DSW]	[mmol kg^-1^ DSW]
Total N	640 ± 90	4.571 ± 0.006
N-NO_3_^-^	4.34 ± 0.81	0.070 ± 0.013
N-NH_4_^+^	2.01 ± 0.47	0.112 ± 0.026
NH_4_^+^:NO_3_^-^ ratio	0.46	1.57

Values presented are the mean (SD). Molar equivalent of each N-related characteristic was calculated basing on the respective weight value. DSW–dry soil weight.

## Discussion

According to our results, it is expected that increasing N deposition in soil causes completion of germination-dependent drift in seedling recruitments. As soil N content rises up to maximal values observed in inland sand dunes, disappearance of *E*. *vulgare*, *H*. *perforatum* and *J*. *montana* can be predicted. Increase in N can cause alternations in micro-habitats, negatively affecting ability to complete germination of *A*. *minima*, *A*. *montanum*, *H*. *arenarium* and *S*. *morisonii* seeds. This leads to the conclusion, that germination requirements of these species are fitted to low N status of poor soils (e.g. Podzols). Similar conclusion was drawn regarding some woodland species from Spain [14] and some species from temperate thermophilous oak forest [40]. Only a few of the species we studied (*C*. *scabiosa*, *C*. *stoebe* and *H*. *radicata*) can efficiently complete germination under high N concentrations, even in 100 mM NH_4_NO_3_ solution. As *C*. *stoebe* [41] and *H*. *radicata* [42, 43] were listed as invasive species, these results partially explain their invasiveness but also show their capability of fast and successful establishment in a wide range of environmental conditions, including inland sand dunes (also those in advanced succession stage). In the case of *C*. *scabiosa*, ability to complete germination in a wide range of conditions observed in our study supports the conception of ‘stress-tolerance’ strategy of this species [44], but probably not inclinations for invasive behavior (due to stable FGP < 50%). It must be however highlighted that data pertaining to multi-species comparisons should be interpreted with caution due to possible dissonance between results of experiments conducted under a given conditions and individual optima of the species. Low germination indices observed in the presented study in several species subjected to control conditions suggest that *in vitro* conditions (physical and/or chemical factors) do not fully match environmental optimum for completion of germination [35].

The most abundant species (frequently only a few) in a given plant community (e.g. psammophilous grassland on acidic soils) can be defined as ‘core species’, while the less abundant ones are ‘satellite species’ [45]. One might expect that species with wide ecological optimum, tolerant to insufficient level of resources or their excess are ‘core species’ and the other ones are ‘specialists’, However, meta-analyses in this field do not support the abovementioned assumption [2]. *J*. *montana*, *H*. *arenarium* and particularly *S*. *morisonii*, the species occurring in substantial numbers in inland sand grasslands [4], showed relatively low completion of germination in the narrow range of conditions, whereas *C*. *scabiosa*, *C*. *stoebe* and *H*. *radicata*, the species persisting on acidic psammophilous grasslands in lesser number [4], showed cosmopolitan reaction in terms of N requirements. This shows that, in extremely poor environments of inland sand grasslands, wide tolerance to environmental N-dependent stimuli at germination stage does not guarantee survival and persistence. As an evolutionary solution to ensure successful establishment in optimal habitats, the seeds of plant species from Asteraceae (e.g. *C*. *scabiosa*, *C*. *stoebe*, *H*. *arenarium*, *H*. *radicata*) are equipped with pappus allowing distribution by wind (anemochory) [46] and by animals (exozoochory) [47] which is beneficial in the open ecosystem. Such an adaptation allows at least some of the produced seeds to colonize more favorable areas.

Our results suggest that *A*. *montanum* does not tolerate NH_4_^+^ as a sole N source, but this effect can be greatly minimized by an equivalent amount of NO_3_^‒^, or even under specific circumstances (10 mM), additional NH_4_^+^ can be beneficial. The presented study strongly suggests meso-stenotopic behavior of this species regarding both N type and dose. Surprisingly, we did not record completion of germination of this species in the control treatment (H_2_O). It is in contrast to the study [48] showing high completion of germination of seeds in distilled water. Such diametrical difference may be due to provenance of seeds, altered environmental conditions affecting development of seeds or to genetic differences between populations from various geographic locations, as it was reported for *Rumex crispus* L. [49]. However, the results similar to ours were presented before [22] regarding the study conducted in 32/20°C thermoperiod in 12/12 h photoperiod. In the same study lower temperature (20/10 and 8/4°C) greatly promoted completion of germination (100 and 56% respectively), which indicates thermoinhibition of germination process in this species. This shows that completion of germination in *A*. *montanum* is under thermal control (this species needs at least cooler night temperature or temperatures characteristic of early spring) but adequate N type and dose substitute for those requirements. Very similar abolition of thermoinhibition by NO_3_^‒^ treatment was presented in the studies on *Lactuca sativa* L. [50]. The authors pointed that the stimulant role of NO_3_^‒^ consisted in induction of NO-dependent signaling transduction pathways and suppression of ABA-dependent regulation of dormancy. This mutual antagonism of NO_3_^‒^-dependent induction of germination and ABA-dependent suppression was widely described before [51]. We can also see strong ecological advantage of this adaptation, as it allows to avoid failure of establishment, e.g. when seeds are deeply buried in sandy soil (low N status [52]) or sown on bare sand (temperature exceeding thermal requirements for completion of germination; even up to 70°C [2]). Our observation can also partially explain why in central Europe (e.g. Poland) *A*. *montanum* can be found on fluvial sands [53], which contain greater amount of nutrients (from alluvial deposits) than aeolic ones [54]. However, *A*. *montanum* is often assigned as a species from alkaline xerothermic grasslands, thus in our opinion habitat preference of this species needs further elucidation.

It was repeatedly showed that increasing N status in soil reduced species richness in semi-arid grasslands [55]. Most species we studied have low or very low N requirements and completed germination mostly at low levels of N, but some completion of germination was also observed in the solutions with N concentrations exceeding environmental values. As it was showed by numerous evaluations, completion of germination under supraoptimal load of N might lead to exhaustion of seed pool in seed bank due to high mortality at seedling stage under mismatched environmental conditions [56]. Among the species we studied only *H*. *perforatum* and *J*. *montana* showed substantial differences pertaining to synchronization of completion of germination (1 mM NH_4_Cl and 1–10 mM of NH_4_NO_3_, respectively). It can be assumed that synchronic completion of germination under such conditions is associated with some kind of adaptation allowing avoidance of the above mentioned seed bank depletion or that it is an efficient mechanism permitting rapid and massive establishment in N-poor vegetation gaps [57]. Completion of germination under very poor conditions suggests that *J*. *montana* is an oligo-stenotopic plant species. Considering this information, germination pattern of *J*. *montana* seems to indicate advanced adaptation allowing successful establishment on inland sand dunes. It was shown in field evaluations, where *J*. *montana* was showed to occur more frequently in low N-deposition sites [58].

Shift from N-poor to relatively N-rich conditions is a natural repercussion of succession in psammophilous grasslands due to deposition of plant biomass [2, 59] which causes gradual development of mineral horizon [9]. Organic forms of N are released from reservoir of plant biomass via microbial metabolism [52] and mineralized throughout succession of acidic grassland [9, 52]. It is worth mentioning that N cycling process in Podzol soils (where mineral forms of N account only 1% of total N [60]) is influenced by many physical and chemical soil traits [2, 52, 61]. It makes hard to predict quantitative equilibrium between NO_3_^‒^ and NH_4_^+^ forms in soil. However, NH_4_^+^ is adsorbed mostly on silt and clay fractions, whilst NO_3_^‒^ remains in a soil solution [62] thus is better available for plants (e.g. during seed imbibition) but is also more likely to percolate to ground water with precipitation [2]. In context of presented results it can be stated that the species persisting in acidic dry grasslands need systematic perturbations uncovering poorer soil layers, free of N depositions.

High light level and light heterogeneity are characteristic of loose-grassland-type vegetation [2, 63]. Light conditions in this type of environments are not strongly differentiated, thus soil seems to be the major factor affecting plant functioning in this community [2]. Perceiving soil nutritional feature through the prism of the N status, we concluded that Entic Podzol, on which the studied community was settled, represented upper values of conditions characteristic of dry acidic grasslands [2]. However, they are still relatively low and indicates poor nutritional quality. It can be even further diminished due to aeolian processes [2]. Furthermore, in this type of environments, microbial nitrification is halted at early succession stages due to low pH of soil and symbiotic N fixation becomes significant only at advanced stages of community development due to establishment of *Trifolium* and *Medicago* species [2]. Due to that, complex transformation of inland sand dunes causes gradual increase in N content in soil up to the stage of succession dominated by grasses, which mechanically stabilize soil, allow development of mineral horizon and deposition of nutrients. This can, however, eliminate species adapted to poor soils.

Summarizing, our study clearly showed that both N type and dose affected the ability of the studied species to complete germination (question 1). Furthermore, each species we studied showed different reaction to N type and dose, which suggests individual requirements for N type and dose to complete germination (question 2). Considering biology of the studied species, it can be seen that plants from dry psammophilous grasslands show two different types of adaptation, i.e. some species are adapted to complete germination on N-poor soils (true pioneer species), whilst only a few of them are able to withstand gradual eutrophication of the occupied habitat. It implies that advancing N deposition on dry psammophilous grasslands (both from natural and anthropogenic sources) may lead to loss of potential niches suitable for propagation of pioneer plants and may reduce genetic and species diversity. Last but not least, influence of progressive homogenization of edaphic conditions on the ability of seeds to complete germination (in terms of increasing N availability) is yet another mechanism that is likely to contribute to secondary succession in dry psammophilous grasslands. Thus, natural as well as anthropogenic processes associated with enhanced N deposition (e.g. due to increased atmospheric N load) shift the environmental balance, favoring establishment of mesophilic species and causing gradual loss of diversity of pioneer plants.

## Supporting information

S1 File Results of experiment. (XLSX)
